# Coordination of carbon assimilation, allocation, and utilization for systemic improvement of cereal yield

**DOI:** 10.3389/fpls.2023.1206829

**Published:** 2023-09-05

**Authors:** Xiao-Gui Liang, Zhen Gao, Xiao-Xiang Fu, Xian-Min Chen, Si Shen, Shun-Li Zhou

**Affiliations:** ^1^ Key Laboratory of Crop Physiology, Ecology and Genetic Breeding, Ministry of Education and Jiangxi Province/The Laboratory for Phytochemistry and Botanical Pesticides, College of Agriculture, Jiangxi Agricultural University, Nanchang, China; ^2^ College of Agronomy and Biotechnology, China Agricultural University, Beijing, China; ^3^ State Key Laboratory of North China Crop Improvement and Regulation, Hebei Agricultural University, Baoding, Hebei, China

**Keywords:** photosynthesis, carbon utilization, sugar transport, systemic signaling, trehalose 6-phosphate, carbon allocation, source-sink relationship, smart crop

## Abstract

The growth of yield outputs is dwindling after the first green revolution, which cannot meet the demand for the projected population increase by the mid-century, especially with the constant threat from extreme climates. Cereal yield requires carbon (C) assimilation in the source for subsequent allocation and utilization in the sink. However, whether the source or sink limits yield improvement, a crucial question for strategic orientation in future breeding and cultivation, is still under debate. To narrow the knowledge gap and capture the progress, we focus on maize, rice, and wheat by briefly reviewing recent advances in yield improvement by modulation of i) leaf photosynthesis; ii) primary C allocation, phloem loading, and unloading; iii) C utilization and grain storage; and iv) systemic sugar signals (e.g., trehalose 6-phosphate). We highlight strategies for optimizing C allocation and utilization to coordinate the source–sink relationships and promote yields. Finally, based on the understanding of these physiological mechanisms, we envisage a future scenery of “smart crop” consisting of flexible coordination of plant C economy, with the goal of yield improvement and resilience in the field population of cereals crops.

## Introduction

Global primary food production needs to double by 2050 to meet the growing demand for food and nutrition (Grassini et al., 2013; Ray et al., 2013). Simultaneously, there is increasing pressure from sustainable development and global climate change, including bioenergy demand, arable land constraints, and extreme weather (Clark et al., 2020; Ortiz-Bobea et al., 2021). Crops in the future will have to be “resilient” and “smart” to cope with unpredictable stresses and thus increase yields in practice. Carbohydrates are pivotal for a crop to balance its maintenance, growth, and yield formation, providing a carbon (C) skeleton, energy substrates, and indispensable sugar signals. Assimilation of C in leaves by light energy conversion export to growing shoots and root systems in specific spatiotemporal patterns is mediated by transporters. Many relevant reviews, based mainly on new findings on carbohydrate transport, sugar sensing, and systemic improvement in model plants, such as *Arabidopsis* and tobacco, have been published (for example, Rolland et al., 2006; Ruan, 2014; Fichtner and Lunn, 2021; Burgess et al., 2023). Maize, wheat, and rice are major food crops worldwide, but their carbohydrate flow and correlated regulation measures have not been well summarized. Here, we briefly review the pathways of C fixation, transport, and storage, mainly in the three cereals, and the key sugar signals that have come to light in systemic regulation during the past few years. We discuss published strategies for the regulation of the plant C economy (“C economy” in this paper refers to the production, circulation, and use of carbohydrates) for crop yield and resilience. We proposed that “systemic enhancement” from source to sink together with “specific optimization” in C allocation management depending on spatial–temporal demand may be the way for both crop yield potential and stress resistance and/or resilience.

## Photosynthesis improvement is showing promise in limited field application

**Figure 1:**
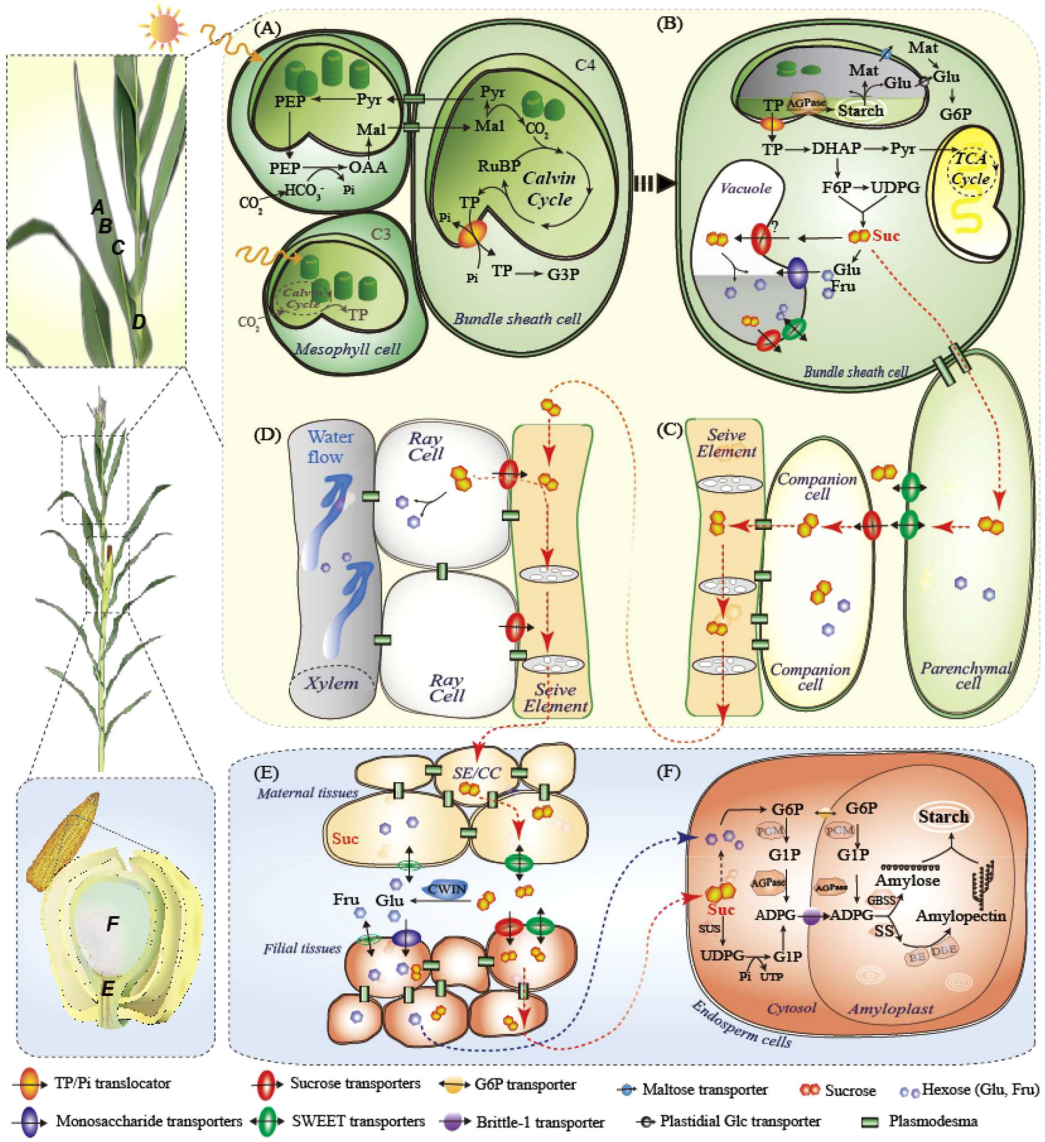
Carbon fixation and sugar flux from leaves to grains, taking maize plants as an example. **(A)** C4 photosynthesis in maize leaves in mesophyll and bundle sheath cells. C3 is also shown in gray in mesphyll cells only. **(B)** Primary carbon partitioning in the diurnal cycle, demonstrating three fates of leaf sugars: glycolysis, temporary storage in chloroplasts or vacuoles, and transport as sucrose; **(C)** Phloem loading of sucrose by SWEET and SUT; **(D)** Sucrose retrieval in vascular; **(E)** Phloem unloading from maternal to filial tissues; **(F)** Starch synthesis in endosperm cells. ADPG, adenosine 5**’**-diphosphoglucose; AGPase, ADPG pyrophosphorylase; BE, branching enzymes; CBC, Calvin Benson cycle; CC, companion cells; CWIN, cell wall invertase; DBE, debranching enzymes; DHAP, dihydroxyacetone phosphate; Fru, fructose; GBSS, Granule-bound starch synthase; Glu, glucose; G3P, glucose 3-phosphate; INV, invertase; ISA, Mal, maleic acid; Mat, maltose; MT, monosaccharide transporter; OAA, oxaloacetic acid; PEP, phosphoenolpyruvate; RUBP, ribulose 1,5-bisphosphate; SE, sieve elements; SS, soluble starch synthase; SWEET, sugars will eventually be exported transporters; SUT, sucrose transporter; SuS, sucrose synthase; PGM, phosphoglucomutase; Pyr, pyruvate; SSS, soluble starch synthase; TCA cycle, tricarboxylic acid cycle; TP, triose phosphate. UDPG, Uridine 5**’**-diphosphoglucose; UTP, uridine triphosphate.

Plants convert light energy into chemical energy *via* photosynthesis (**Figure 1A**). The theoretical maximum efficiencies of photosynthetic energy conversion are approximately 4.6% and 6% for C3 and C4 plants, respectively, as estimated using biomass (Zhu et al., 2010). However, efficiency in the real world, even under favorable conditions, is only half or less than the theoretical value (Yin and Struik, 2015). As a well-studied pathway, the limitations of photosynthesis have been modeled. The improvement of the photosynthetic system is mainly projected into three phases: near-term (including photorespiration bypass, canopy structure improvement, RuBP regeneration, and chlorophyll optimization), mid-term (including photoprotective recovery and RubisCO carboxylation improvement), and long-term (including RubisCO oxygenase decline, mesophyll conductance, and conversion of C3 to C4) (Zhu et al., 2010; von Caemmerer and Furbank, 2016; Leister, 2023).

In the past two decades, researchers have proposed yield-improvement strategies based on enhanced photosynthesis using synthetic biology or gene editing in *Arabidopsis* and tobacco (Long et al., 2018; Hart et al., 2019; Papanatsiou et al., 2019; Chen et al., 2020; Lopez-Calcagno et al., 2020). A few recent studies have shown promise for field crops. One synthetic photorespiratory pathway boosted tobacco biomass by up to 40%, whereas another led to an observable increase in photosynthesis and grain yield under high light in field-grown rice plants (Shen et al., 2019; South et al., 2019). Transgenic rice overexpressing a RuBisCO subunit improved yield performance and nitrogen (N) use efficiency for biomass production when receiving sufficient N fertilization in an experimental paddy field (Yoon et al., 2020). The total spikelet number of transgenic rice did not change, but the ratio of filled spikelets increased, resulting in a 20%–28% higher yield than the wild type (Yoon et al., 2020). In *Arabidopsis* and soybean, a large increase in grain yield under fluctuating light conditions was achieved by accelerating the recovery from photoprotection. However, when lodging (caused by storms) and/or reduced cloud cover resulted in a lack of sun-flecks in the canopy, the yield gain brought by genetically modified soybeans disappeared (Kromdijk et al., 2016; De Souza et al., 2022). These examples show that increases in photosynthetic efficiency can improve crop yield, under nutrient availability or specific light conditions. However, under adverse conditions such as drought or barren, which occur frequently around the world today, the yield gains shrink or disappear. As Sinclair et al. (2019) refuted, photosynthesis depends on N (and other nutrients). When nitrogen is reduced, plants with high photosynthetic efficiency may not increase crop yield but will cause resource competition and waste, further leading to insufficient transport of sugars into sink tissues. Cross-scale model studies validated that under water-limited conditions, high photosynthetic efficiency could lead to early consumption of soil water and later-growth-period drought and reduce crop yield; however, under water-abundant conditions, improvement of RubisCO and SeBP increased the yield of wheat and sorghum in Australia (Wu et al., 2019a; Wu et al., 2023). In contrast, elevated CO_2_ and phosphate pools have been modeled to synergistically enhance C3 photosynthesis (Khurshid et al., 2020).

Scholars engaged in photosynthetic efficiency research have notice that proper field nutrition management and coordinated plant C economy are critical to yield as photosynthesis. Transgenic rice plants with modified photorespiration and enhanced photosynthesis undergo massive grain abortion, consistent with a marked reduction in sugar transport from source to sink, as tracked by ^13^C isotope labeling (Wang et al., 2020). Therefore, sustainable C economic growth of plants is proposed here to depend on further timely and reasonable allocation and utilization after photosynthesis enhancement.

## Primary C allocation in photosynthetic leaves indicates sink growth and resilience

While photosynthesis occurs only in light, growth and respiration occur throughout the day–night cycle (Smith and Stitt, 2007). The immediate photo-assimilate is partitioned into a fraction for glycolysis consumption, a fraction for sucrose transport, and a fraction for temporary storage in leaves and remobilized during the night (**Figure 1B**). In *Arabidopsis*, starch is the main transitional reserve in leaves (up to 50% or more) and is synthesized and degraded linearly in a diurnal cycle to maximize C utilization and prevent starvation in changing environments (Stitt and Zeeman, 2012; Ruan, 2014). Furthermore, precisely controlled starch turnover has been shown to be negatively correlated with growth rate or biomass accumulation (Cross et al., 2006; Sulpice et al., 2009; Sulpice et al., 2014), reflecting that the more C reserved in leaves, the less sink obtained for growth. Similarly, the ratio of daily starch accumulation to net C assimilation is negatively correlated with the ground biomass and final yield of three representative maize hybrids (Liang et al., 2019). Under low light or prolonged darkness, maize leaves allocate an even lower proportion of reduced photosynthetic products to sink ends, such as the developing ears, leading to biomass and/or yield losses (Liang et al., 2020; Liang et al., 2021). Thus, it may be possible to increase crop yield potential and resilience by allocating more primary C to sinks under certain circumstances (Oszvald et al., 2018). However, compared to the starchy leaves in *Arabidopsis*, crops such as wheat and rice prefer soluble sugar leaves, while maize leaves are intermediate (Smith and Stitt, 2007; Liang et al., 2021), the role of C storage in cereal leaves deserves further validation.

Some transporters are directly responsible for the primary C allocation. Sucrose transporter 2 (SUT2), located on the vacuolar membrane, transiently stores sucrose for subsequent growth in cereals (Leach et al., 2017; Prasad et al., 2023). Both loss-of-function mutants of o*ssut2* and *zmsut2* exhibit severe growth restriction and accumulate more sucrose, fructose, glucose, and starch in the leaves (Eom et al., 2011; Leach et al., 2017). Rice cultivars with increased yield under elevated CO_2_ conditions exhibited elevated expression of *OsSUT1* and *OsSUT2* and increased photosynthetic capacity of flag leaves, suggesting that enhanced export can prevent inhibition of photosynthesis by sugar accumulation (Zhang et al., 2020a). Other transporters that control sugar transport across vacuole in mesophyll cells, such as the tonoplast monosaccharide transporters (TMTs, TMT1, 2), the class IV sugars will eventually be exported transporters (SWEETs, SWEET16, 17), has also been shown to regulate plant growth and stress resistance in several species (Wingenter et al., 2010; Liu et al., 2022b; Zhu et al., 2022). But as far as we know, related studies on major cereal crops are rare.

In summary, our preliminary findings indicate that daily C turnover in expanded leaves is directly related to crop growth and resistance. Boosting diurnal leaf sucrose export appears to be a strategy to improve cereal yield or stress tolerance in sink tissues, at least in some cases. However, it remains unclear whether this sacrifices the ability of the source leaves to survive extreme stress with fewer sugar reserves. The primary C allocation characteristics and underlying physiological mechanisms in different crops and varieties require further investigation.

## Phloem loading and unloading: the linkage between leaves and sinks

Long-distance sugar transport requires phloem loading and unloading to coordinate leaf C supply and sink growth in different environments. Phloem tissue is composed of three cell types: companion cells (CC), sieve elements (SE), and phloem parenchyma cells (PP), which act as highway linking sources and sinks for sugar transport. Proteins responsible for sugar transport, including SUT, SWEETs, and monosaccharide transporters (MTs), have receive much attention (Julius et al., 2017; Braun, 2022; Xue et al., 2022; Singh et al., 2023). Here, we focus on maize, rice, and wheat and discuss the importance of the coordination of C transport for crop production in some recent cases.

Sucrose, the principal sugar for transport, is produced in mesophyll cells (C3) or bundle sheath cells (C4) and moves into adjacent phloem parenchyma cells through plasmodesmata (symplastic movement). In the subsequent apoplastic transport, sucrose is excreted into the intercellular space by clade III SWEETs and collected by SUT1, which is located on the plasma membrane of CC cells against the concentration gradient in the CC–SE complex (**Figure 1C**; Xue et al., 2022). Mutants of *Zmsut1* and *Ossut1* are severely debilitated in growth and grain filling (Scofield et al., 2002; Slewinski et al., 2009). However, both mutants could grow to maturity and produce fertile seeds, suggesting that sugars could be transferred by other SUTs (perhaps *OsSUT5* in rice, see Wang et al., 2021b) or paths. Through hydrostatic pressure established in the phloem, sucrose is transported to the sink organ by the SE, where sucrose leakage is retrieved by SUT1 during long-distance transport (**Figure 1D**; Ohshima et al., 1990; Knoblauch et al., 2016). Sucrose unloading occurs symplasmically in growing radicles and shoot apices, while in cereal grains, SWEETs, SUT1, cell wall invertases (CWINs), and MTs are required for maternal-to-filial transport (**Figure 1E**; Apoplastic path, see details below; Haupt et al., 2001; Dhungana and Braun, 2021; Ruan, 2022; Shen et al., 2022).

SWEETs may be among the most complex sugar transporter families (Chen et al., 2012; Eom et al., 2015; Xue et al., 2022; Singh et al., 2023). The number of SWEETs are 24, 21, and 59, while the SUTs are 5, 5, and 18 in maize, rice, and wheat, respectively (Zhu et al., 2022; Prasad et al., 2023). In maize, SWEET13, including *ZmSWEET13a*, *b*, and *c*, is responsible for sucrose efflux to the SE–CC complex. The triple-knockout mutants exhibited similar but milder growth to *Zmsut1*, implying greater genetic redundancy among clade III SWEETs (Bezrutczyk et al., 2018). Similarly, *OsSWEET11, 13*, *14*, and *15* are expressed in the rice leaf phloem and are thought to play a role in phloem loading (Yuan et al., 2014; Eom et al., 2019; Mathan et al., 2021a). Single-knockout mutants of *Ossweet 11*, *13*, and *14* showed milder or no yield penalty, whereas double-knockout mutants of *Ossweet11* and *14* had severe phenotypes (Eom et al., 2019; Fei et al., 2021). Blocking sugar transmembrane loading by overexpressing CWIN or by knocking down *OsDOF11* (DNA binding with one finger 11), which binds and activates gene expression of *OsSUT1*, *OsSWEET11*, and *OsSWEET14*, resulted in restricted vegetable growth and decreased grain yields (Wu et al., 2018; Wang et al., 2021b). In contrast, enhanced apoplastic phloem loading under low nitrogen conditions was attributed to increased gene expression of *OsSUT1*, *OsSWEET11*, and *OsSWEET14* in leaves and stems (Li et al., 2022a). Furthermore, the lack of symplastic connection between the SE–CC complex and surrounding parenchyma cells in leaves and stems was verified by the phloem-mobile symplastic tracer carboxyfluorescein (Li et al., 2022a).

Both SUTs and SWEETs are believed to have undergone post-domestication selection for higher-caloric harvests (Sosso et al., 2015; Mathan et al., 2021a; Singh et al., 2023). Researchers have attempted to modify the expression of SUT1 and/or clade III SWEETs to coordinate C transport. However, unexpectedly, constitutive overexpression of *OsSWEET11*, *OsSWEET14*, *OsDOF1*, *OsSUT1*, and *OsSWEET11* and *14* in rice resulted in attenuated growth and yield penalty, similar to the *AtSWEET11* and *12* OE lines in *Arabidopsis* (Gao et al., 2018; Kim et al., 2021; Singh et al., 2021; Fatima et al., 2023). Surprisingly, the *OsDOF11* and Os*SWEET 14* OE lines showed improved resistance to plant pathogens, such as *Xanthomonas oryzae pv oryzae* and *Rhizoctonia solani*, which are known to induce the expression of SWEETs for sugar secretion and nutrition hijacking (Eom et al., 2019; Oliva et al., 2019; Wu et al., 2022). Constitutive overexpression of SWEET may induce a series of plant defense reactions, leading to a trade-off between growth and resistance (Xue et al., 2022). Thus, specific regulations are more reasonable. Field rice plants overexpressing *AtSUT2* under the control of a phloem-specific promoter, showed a 16% increase in grain yield (Wang et al., 2015). Tissue-specific activation of *OsDOF11* increases both yield and resistance to *R. solani* (Kim et al., 2021). More ingeniously, by creating genomic mutations in the SWEET (*OsSWEET11*, *13*, *14*)-specific promoter, where pathogen-secreted transcription activator-like effectors bind to induce gene expression, endowing rice lines with robust, broad-spectrum resistance (Eom et al., 2019; Oliva et al., 2019).

Sugar loading and unloading clues and the significance of these transporters in improving crop performance remain to be explored. First, new sugar transporters are yet to be discovered, although it has been suggested that most sugar transporters have been identified (Chen et al., 2015). Recently, a nitrate transporter 1/peptide transporter family member named *ZmSUGCAR1* was shown to carry both sucrose and glucose for grain filling and was proposed to be conserved in wheat and sorghum (Yang et al., 2022). Whether other nitrate/peptide transporters in this family are involved in sugar transport and whether substrate competition affects transporter selectivity is unclear. Second, the substrate selectivity of sugar transporters in crops and their correlation with growth and abiotic stress resistance need to be explored. Clade III SWEETs and clade I SWEET3a glucose transporters can transport gibberellin hormones in addition to sugars (Morii et al., 2020; Wu et al., 2022). *OsSWEET13* and *15* were strongly expressed under drought, salt, and ABA treatment, which revealed that the ABA-responsive transcription factor *OsbZIP72* directly binds to the promoters of *OsSWEET13* and *15* and activates their expression, likely to improve the root-shoot ratio for higher tolerance (Mathan et al., 2021b; Chen et al., 2022a). Third, functional redundancy within the family or clade and interactions among different types of sugar transporters are largely unknown. In general, exploration of the above issues would further deepen our understanding of the critical role of sugar transporters in coordinating the plant C economy and simultaneously improving crop yield and resilience.

## Phloem unloading: how sugar transport from maternal to filial tissues determines crop yield

Besides the endosperm and embryo, cereal grains also comprise multiple distinctive or even transgenerational tissues, such as the maternal placentachalaza in maize, filial basal endosperm transfer cells (named endosperm transfer cells in wheat), and embryo-surrounding region (ESR) (see details in Liu et al., 2022a; Shen et al., 2022). Within developmentally specific but functionally coordinated tissues, sugar transporters and CWIN set a typical manifestation in which their locations mandate functions in phloem unloading and determine grain development. We recently built a holistic view of sugar transporters that control sucrose unloading in maize grains (Shen et al., 2022). *ZmSWEET11* and *13b* located in the placento-chalazal zone, expel sucrose into the apoplasmic space and, *ZmSUT1*, *ZmSWEET11/13a* (sucrose transporters), and *ZmSTP3*, *ZmSWEET3a/4c* (monosaccharide transporters), located in the basal endosperm transfer cells, retrieved sucrose or hexoses after hydrolysis by CWIN (**Figure 1E**). Sucrose could be further transported by the embryo-surrounding region (ESR) located in *ZmSWEET14a/15a*, broken down by the ESR-embryo junction located in CWIN, and retrieved by embryo-located *ZmSUT4* for embryo development (Shen et al., 2022). Sucrose synthase (SUS) and invertase are responsible for sucrose cleavage in the endosperm and embryo. Similarly, sucrose or monosaccharides derived from *GIF1* (also named *OsCWIN2*) in the vascular bundle are transported by *OsSWEET11*, *14*, and *15* in rice. *OsSUT1*, *3*, and *4*, *OsSWEET4*, *11*, and *14*, as well as possibly *OsMT4* and *6*, are responsible for transporting sugar to the aleurone layer for grain growth and C storage (Ma et al., 2017; Yang et al., 2018; Solomon and Drea, 2019; Fei et al., 2021; Li et al., 2022b; Liu et al., 2022a). Although the assimilate acquisition route in wheat differs that from rice and maize (Solomon and Drea, 2019; Liu et al., 2022a), the transporters responsible for apoplast transport may be similar. A recent study showed that TraesCS4B02G287800 and TraesCS4D02G286500 (homologous to *OsSUT1*), and TraesCS2D02G293200 and TraesCS2B02G311900 (homologous to *OsGIF1*) are involved in low-light induced sugar transport in wheat grains (Yang et al., 2023).

CWIN is proposed to play a major role in early grain development, probably promoting glucose-activated nuclear division for large endosperm capacity and embryo fertility (Ruan, 2014; Ruan, 2022). Both mutants of *Mn1* (also named *ZmCWIN2*) in maize and the ortholog gene *OsGIF1* in rice exhibited reduced grain size, indicating the irreplaceable roles of hexose supply and sugar signaling generated by CWIN (Wang et al., 2008; Li et al., 2013). Ubiquitously expressed *Mn1* has the highest expression in developing maize seeds, specifically at the grain set stage (Li et al., 2013). Constitutive overexpression of *AtGIF1*, *OsGIF1*, or *Mn1* in the maize inbred line Ye478 results in increased grain number, grain weight, starch content, and final yield (Li et al., 2013). In rice, 35S or *Waxy*-promoted ectopic expression of the *OsGIF1* gene showed small grains similar to the *gif1* mutant, but the native promotor-driven Os*GIF1* increased yield production (Wang et al., 2008). The interactions between CWIN and sugar transporters remain largely unknown. CWIN is co-expressed with hexose transporters located at the plasma membrane of sinks. Sosso et al. (2015) proposed that SWEET4-mediated hexose transport acts downstream of a CWIN in maize and rice. Both *ZmSWEET4c* and *OsSWEET4* mutants are defective in seed-filling (Sosso et al., 2015).

Sugar transporters and invertases in phloem loading and unloading are essential for yield. However, the specific function of each transporter and its responses to C availability (and external stimuli) need to be elucidated. During drought-induced kernel abortion, C shortage suppressed ZmSWEET effluxers (located on the PC and ESR), CWIN, and SUS, but stimulated ZmSTPs and ZmSUTs, which are responsible for sugar uptake in filial tissues (Shen et al., 2020; Shen et al., 2022). When the C supply was boosted, the doomed kernels were reformed, and drought-induced changes in the transporters were mostly prevented (Shen et al., 2022). Sugar signals may regulate transporters and their coordination; however, the specific mechanisms remain unclear. In addition, how these proteins respond to other signaling pathways, such as phytohormones, remains largely unknown.

## Regulation of starch synthesis increases sink demand and crop yield

After maize kernel capacity was established, CWIN expression was repressed and sucrose was directly transported into the endosperm in maize, where SUSs such as shrunken1 (*ZmSh1*) and sucrose synthase 1, 2, and 4 (*ZmSUS1/2/4*) were highly expressed during the grain-filling stage (**Figure 1F**; Larkins, 2017; Shen et al., 2022). Starch is the main storage site in the cereal endosperm, accounting for more than 70% of the endosperm dry weight. Starch synthesis is highly regulated by enzymes including adenosine diphosphate (ADP)-glucose pyrophosphorylases (AGPases), soluble starch synthases (SSs), granule-bound starch synthases (GBSSs), starch branching enzymes (BEs), and starch debranching enzymes (DBEs) (Jeon et al., 2010). Interestingly, the order of starch accumulation in different parts of one grain is highly conserved in maize, rice, and probably wheat, starting from the distal end of the sugar unloading position and gradually moving to the proximal end (Chen, 2022; Liu et al., 2022a). Sucrose, but not hexose, is thought to be resynthesized at the base of the maize endosperm (the site of sugar unloading) and transported to the site of starch synthesis, which is inconsistent with the substantial upregulation of SUSs during grain filling (Shannon et al., 1986; Olsen, 2020; Shen et al., 2022). AGPase is a key enzyme in starch synthesis. Overexpression of AGPase or SS has been reported to increase cereal grain weight, starch content, and yield (Smidansky et al., 2002; Tian et al., 2018; Paul et al., 2020). A recent study engineered heat-stable 6-phosphogluconate dehydrogenase in maize to improve grain yield under heat stress (Ribeiro et al., 2020). By altering these critical metabolic enzymes, yield performance can be improved by increasing sink demand; however, further efforts are needed in field applications.

## Systemic sugar signaling regulates C partitioning

Many of the details related to sugar sensing, signaling, and crosstalk with phytohormones and environmental nutrients are largely performed in model plants (Wu et al., 2019b; Baena-Gonzalez and Lunn, 2020; Fichtner et al., 2021; Li et al., 2021; Meng et al., 2022), while for cereals, the understanding of sugar signal transduction and regulation is still insufficient. Here, we briefly introduce the core networks of sugar sensing and signaling. Specifically, recent examples of the regulation of trehalose-6-phosphate in cereal crops are discussed, with the aim of revealing the potential of systemic regulation to coordinate source-sink C balance and synchronously enhance crop yield and resilience.

**Figure 2:**
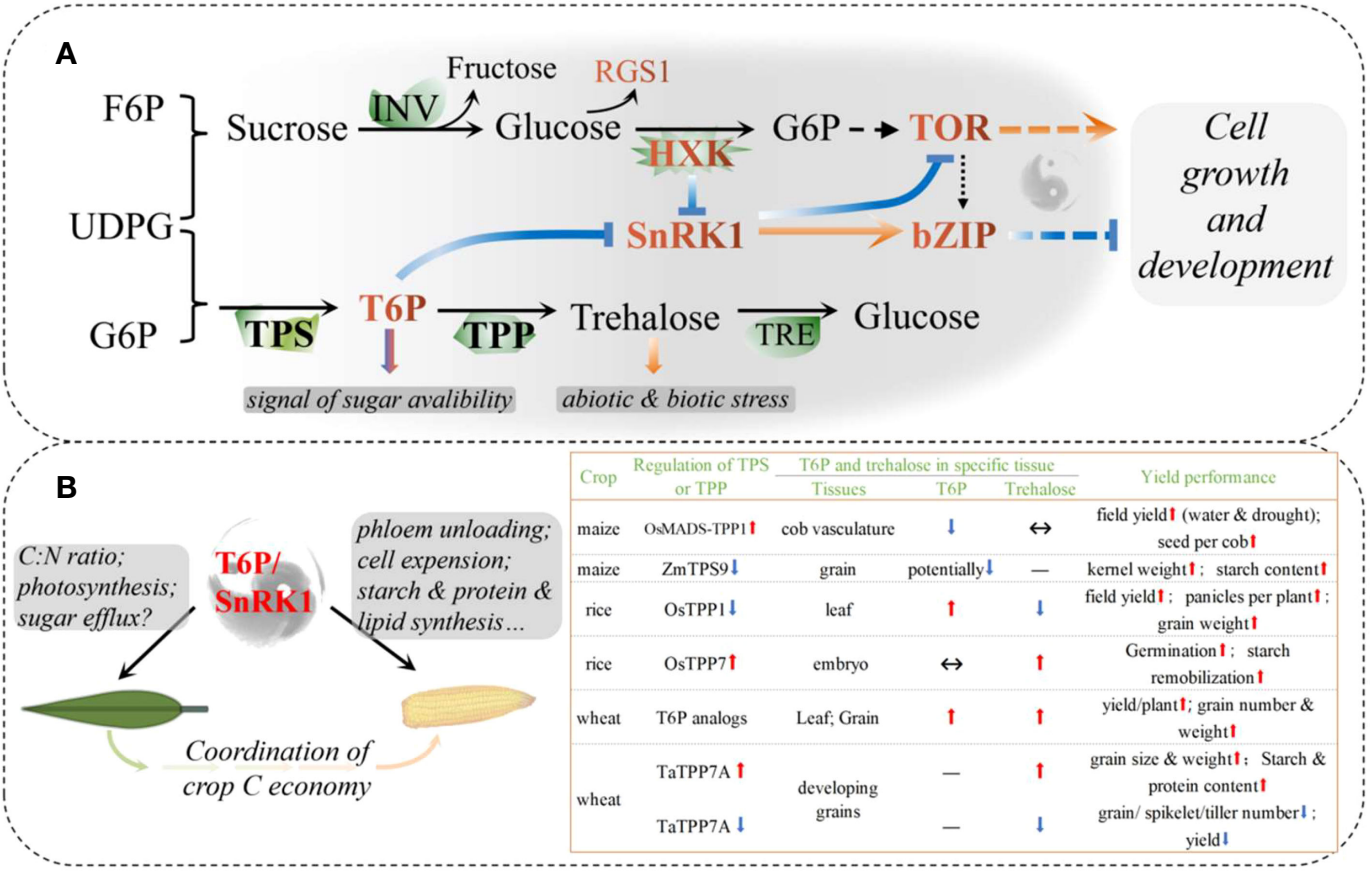
Systemic sugar signals. **(A)** Interactions in core sugar sensing and signaling; **(B)** Schematic diagram of source-sink carbon balance regulated by a possible T6P/SnRK1 complex in cereals. Recent reports on the regulation of T6P signaling in maize, rice, and wheat have been summarized. bZIP, The basic leucine zipper domain; F6P, fructose 6-phosphate; G6P, glucose 6-phosphate; HXK, hexokinase; INV, invertase; RGS1, regulator of G-protein signaling1; SnRK1, sucrose non-fermenting related kinase 1; T6P, trehalose 6-phosphate; TOR, target of Rapamycin; TPP, T6P phosphatase; TPS, T6P synthase; TRE, trehalase; UDPG, Uridine 5**’**-diphosphoglucose.

There are two main mechanisms for sensing and transducing sugar signals in plants, called: direct and indirect (**Figure 2**; Li et al., 2021). The former is triggered by sugar-binding sensors, such as the glucose signaling sensor hexokinase (HXK), and possibly the regulators of G-protein signaling1 (RGS1), trehalose 6-phosphate (T6P) synthase1 (TPS1), and T6P phosphatase (TPP). The latter includes sugar-derived bioenergetic molecules and metabolite-regulated signaling proteins, such as the glucose-activated target of rapamycin (TOR) and sugar-inhibited SNF1-related protein kinase 1 (SnRK1). HXK1 controls multiple biological processes, including photosynthesis, phytohormone production, growth, and senescence, which are uncoupled from sugar metabolism (Moore et al., 2003). T6P, known as plant “insulin” is a key signal indicating sucrose availability and regulating sucrose homeostasis systemically (Fichtner and Lunn, 2021). TOR kinase acts as a GPS of nutrient, energy, and environmental cues to orchestrate growth and development, whereas SnRK1 antagonizes TOR by phosphorylating RAPTOR, a subunit of the TOR complex (**Figure 2A**). The SnRK1 complex plays a central role in nutrient sensing and stress responses and is activated under nutrient deprivation, such as darkness and starvation, but is inhibited by sugar phosphates, such as glucose 1-phosphate and T6P (Zhang et al., 2009; Li et al., 2021). By downregulating anabolism and upregulating catabolism, SnRK1 restores cellular energy homeostasis and coordinates tissue response to the environment.

T6P is an intermediate in the trehalose biosynthesis pathway mediated by TPS1 and TPP. As a signal and regulator of sucrose status, T6P functions, at least partly, if not all, through the inhibition of SnRK1, and trehalose has long been implicated in plant biotic and abiotic stress responses (Baena-Gonzalez and Lunn, 2020). Recent studies on the regulation of T6P have shown promising results in major crops, with a significant increase in both yield and stress resistance or resilience (**Figure 2B**; Paul et al., 2018). In maize, floral promoter *OsMads6* driven overexpression of the rice TPP1 gene in developing maize ears improved yield under both drought and non-drought conditions over multiple field sites and seasons (Nuccio et al., 2015). *OsMads6* is most active in phloem CC cells in florets and piths, leading to the largest decrease in T6P levels, but significantly increased expression of SWEETs in these tissues. Hence, an increase in both sucrose in ear spikelets and photosynthesis in leaves during the flowering period can be explained by boosted phloem unloading (Nuccio et al., 2015; Oszvald et al., 2018). A common drought-induced phenomenon in maize production is the abortion of apical kernels, where the expression of TPS is elevated, but TPP is lower when compared to set kernels. Abortion can be largely rescued by synchronous pollination and/or incomplete basal pollination, accompanied by downregulation of TPS and upregulation of TPP expression (Shen et al., 2018; Shen et al., 2020). *ZmTPS9* was recently identified as a non-starch pathway gene that contributes to starch synthesis. Gene editing to knockout *ZmTPS9* results in increased starch accumulation and kernel weight (Hu et al., 2021).

The contributions of T6P, TPS, and TPP genes to source- and sink-related traits were further confirmed by gene-based mapping and T6P-precursors in wheat (Griffiths et al., 2016; Lyra et al., 2021). Using plant-permeable analogs and sunlight-triggered release of T6P, a chemical intervention was proposed to increase wheat grain yield spraying during grain filling and to prevent drought during the vegetative stage (Griffiths et al., 2016). However, unlike maize, the increased yield was associated with increased T6P in wheat grains, suggesting that the role of T6P may differ among species. Another possibility is that T6P has different effects on the source and the sink. The sprayed T6P-precursors to the ears only or to the whole plant cannot enter the grain without affecting other tissues, although the T6P in the grain was elevated (Griffiths et al., 2016). T6P in other parts (including leaves, glumes, and vasculature) is not known, and there may be huge differences in the pericarp and endosperm in one grain owing to unclear transport characteristics. T6P-precursors spraying not only increased gene expression related to the cell wall, starch, and protein synthesis in grains, and increased sugar availability in new-born leaves after post-drought spraying, but also increased photo-assimilation and preserved less C in source tissues, including flag leaves, aging leaves, and potentially pericarps (Liang, 2019). As a systemic signaling pathway, the role of T6P in cereal crops may be similar, but there are existing spatiotemporal differences.

In general, we propose that the increase in T6P could accelerate sucrose transport and/or conversion in source tissues (here, where net C outcomes above zero are defined as “source”) and hence boosts photosynthesis in leaves, while increased sugar accumulation in the sink tissues (where net C incomes above zero are defined as “sink”) could be associated with decreased T6P (**Figure 2B**). Consistent with this speculation, *TaTPP-7A* was detected as a QTL that was significantly associated with grain weight, and overexpression of *TaTPP-7A* greatly enhanced grain weight and wheat yield (Liu et al., 2023). Overexpression of *OsTPP7* located in coleoptile tips enhanced rice germination under both anaerobic stress and an aerobic environment by stimulation of endosperm starch remobilization, whereas *Ostpp1* mutants germinated slower than the wild type (Kretzschmar et al., 2015; Wang et al., 2021a). Recently, a sugar-inducible rice transcription factor, *OsNAC23*, was found to directly repress *OsTPP1* expression to simultaneously elevate T6P, thereby facilitating C partitioning from the source to sink. Plants overexpressing *OsNAC23* showed elevated T6P levels, sugar transport, and photosynthesis in flag leaves and increased sink organ size and rice yields in three elite-variety backgrounds and two locations (Li et al., 2022c). These results further confirmed our speculation and showed promise for future T6P modulation of both yield potential and resistance/resilience (**Figure 2B**). Future research may need to explore different strategies to regulate T6P in sources and/or sinks and to further clarify the roles of different TPSs and TPPs in the processes of yield production and resilience.

## The source-sink system: the Yin–Yang balance

High photoassimilation is the basis for yield and resistance only if the carbohydrates can be effectively stored or transported in downstream processes. First, triose phosphate (TP) from the Calvin cycle must be exported from the chloroplast into the cytoplasm in exchange for inorganic phosphate (Pi). High TP in chloroplasts results in high levels of 3-phosphoglyceraldehyde, low Pi, and photosynthetic inhibition, thereby activating AGPase and starch synthesis. However, a low TP leads to the opposite (Mugford et al., 2014). Starch accumulation in leaves can relieve photosynthetic inhibition but is negatively correlated with maize growth (Liang et al., 2019; Liang et al., 2020). Hence, increasing P availability could be a feasible way to improve the yield of photosynthetically improved crops (Khurshid et al., 2020). Second, excessive soluble sugars in the cytoplasm also feedback-inhibit photosynthesis, which could be alleviated by TMT-mediated temporary storage in the vacuole or by an efficient transport system that is co-controlled by SWEETs and SUT1 in leaves. Third, stems have multiple roles in carbohydrate coordination, acting as a transfer tissue, temporary sink, and donator as needed (Slewinski, 2012). Regulation of stem sugars has been shown to boost yield and resilience as early as the first green revolution. Finally but most importantly, sugars loaded into the phloem must be utilized promptly and appropriately. Sugar unloading strength and sink growth activity ultimately determine the persistence of the source strength.

**Figure 3:**
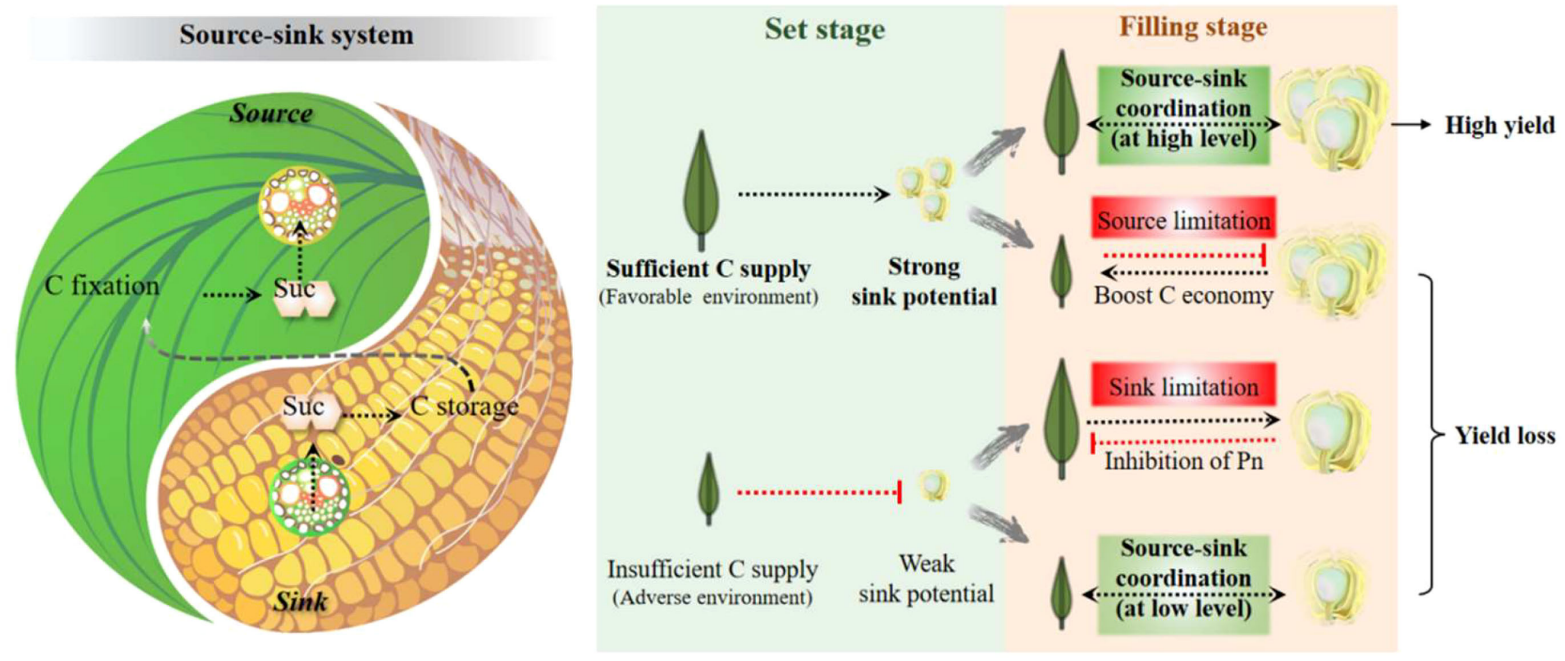
The dialectical relationship between source and sink from a systemic perspective with the example of carbohydrates. This relationship is described as the balance of Yin and Yang, the wisdom crystallization of ancient Chinese. The different scenarios of the source-sink relationship during the grain set and filling stages are briefly summarized. It is proposed that the C supply from the source determines the sink capacity during the grain-set stage, whereas in the grain-filling stage, the realization of a potentially high yield requires a high-level balance between the source and sink. C, carbon; Suc, sucrose.

There is still no verdict about the debate on whether cereal yield is source or sink-limited. Researchers focusing on the source (e.g., photosynthesis system) and sink (e.g., sugar unloading and utilization) both believe that their work could solely improve yield output, while usually reaching an opposite or unexpected result. The so-called “source limitation” or “sink limitation” is more a matter of, at least partly, synergies between organ growth, phase transition, and environmental changes. As mentioned above, the flow of C (and other nutrients) in plants determines that the development of the source-sink relationship always maintains a dynamic balance, rather than mutual independence or even antagonism. The status between the source and sink can be described as Yin and Yang in Tai Ji, the two opposing and unifying principles in nature (**Figure 3**). Uncoordinated relationships in the crop are generally divided into two cases, i.e., sufficient source supply with insufficient sink demand and the opposite, in which the plant as a whole system tries to turn to but probably never attains a balance. There is no doubt that the strengthening or weakening of one can pull or feedback inhibits the development of the other. For example, evaluated leaf photosynthesis by CO_2_ concentration increased yield performance, whereas a larger sink capacity facilitated higher power of C fixation and higher ratios of transfer (Arp, 1991; Paul and Foyer, 2001; O'Leary et al., 2015).

The question is, will the improvement of one be sufficient to stimulate the other to achieve a high level of source-sink balance? A combined approach using both ‘pull’ and ‘push’ has been reported for yield improvement (Rossi et al., 2015). The long time-scale adaptation of the crop to the environment makes it difficult to bring out ideal results by one or two gene modifications, although silencing or knockout of particular genes has resulted in phenotype defects. Metabolic engineering also suggests that an optimal balance of enzyme activities is more important than simply overexpressing a suite of enzymes (Sweetlove et al., 2017). Therefore, rather than improving only the source or sink, systemic improvement and whole-plant C balance may be more important for crop production. This can be achieved by introducing multiple targeted engineered genes from both the source and sink tissues, such as transporters, critical enzymes, and systemic signals, into crops.

## Optimizing plant C economy for both yield and resilience

Empirically, crop yield and stress resistance often contradicts each other. However, by optimizing the plant C economy, several genetic strategies have emerged that can increase crop tolerance to stress while increasing, or at least not reducing, yield. Editing of the SWEETs promoter mentioned above is a precise strategy to improve disease resistance while maintaining functional SWEETs for crops (Eom et al., 2019; Oliva et al., 2019). ABA is a key response signal for abiotic stresses such as drought. Sustained ABA signaling is considered to significantly increase plant resistance but at the expense of a growth penalty. Overexpression of ABA signaling receptors (TaPYLs), which can respond rapidly to drought-induced ABA signals, improves wheat yield under drought conditions without affecting non-drought growth and yield (Mega et al., 2019). Overexpression of the brassinosteroid receptor BRL3 confers drought resistance without affecting plant growth (Fàbregas et al., 2018). Trehalose accumulates under various conditions and protects plants from damage. Researchers have used fusion gene coding for TPS and TPP driven by an ABA-inducible promoter to generate transgenic rice. Trehalose overproduction contributes to lower yield penalties under drought, saline, and sodic conditions, while the yield potential remains unchanged (Joshi et al., 2020). These examples, together with the above-mentioned T6P modulation are not representative of all, but raise the point of view that carbohydrates can be allocated to the right place at the right time by spatiotemporal expression of certain gene(s) through manipulation of specific promoters (conditionally induced or tissue-specific). This flexible C economy strategy could simultaneously endow crops with both high-yield traits and good resistance/resilience.

In field production, several strategies for simultaneously improving crop resistance and yield by targeting C allocation have gradually emerged. Ovary or grain abortion is an adaptive response in cereal domestication, but is agronomically undesirable as it prevents crop yields from reaching their potential. The window period for ovary pollination and grain establishment is considered highly sensitive to stress (Shen et al., 2018; Gao et al., 2023). Shortening maize ASI in the past has improved grain yield due to, at least partly, increased C allocation into the ear tissue. Furthermore, the pollination time gap (PTG) of ovaries on different parts of a panicle/cob will lead to uneven distribution of C allocation among grain siblings, resulting in abortion of inferior spikelets, which usually occur at the top of maize ears, the upper and lower parts of rice, and wheat spikes, especially under adverse conditions such as drought (Shen et al., 2018; Shen et al., 2023). Shortening PTG by measures, such as synchronizing pollination or adoption of stubby ear hybrids, has been shown to coordinate C allocation within siblings and improve both yield and drought resistance in maize (Shen et al., 2018; Shen et al., 2020; Chen et al., 2022b). Strategies aimed at shortening PTG for rice and wheat might work equally well. Moreover, increased distribution of C in the ovary or grain can be achieved by stem manipulation. In wheat, drought-induced abortion of inferior ovaries or grains is associated with suppressed ABA signal transduction in the stems (Zhang et al., 2020b). In contrast, moderate post-anthesis drought in rice-induced ABA signaling and ABA–IAA interactions promotes the remobilization of stem-stored C reserves and enhances inferior grain filling (Wang and Zhang, 2020; Teng et al., 2022). Similarly, recent research has highlighted ways to increase maize grain number and final yield under both optimal and unsuitable environments by tuning stem elongation and ear development. Two maize genotypes with similar plant heights and yield potentials but different drought tolerances were subjected to water scarcity. The ear grain number and final yield of the tolerant genotype were 38.1% and 35.1% higher, respectively, but the plant height was 17.6% short than that under drought (Gao et al., 2023). ^13^C labeling, together with transcript analysis revealed that the inhibited stem elongation and promoted assimilate allocation to the ear in the tolerant hybrid were induced by signals including ABA and T6P in the stem (Gao et al., 2023). Exogenous application of plant growth regulators, such as ethephon and cycocel at the V15 stage of maize hybrids was proved to reduce internode length and facilitate assimilate partitioning to the ear, which in turn increases the final yield (Zhao et al., 2022). In general, as evidenced by the first green revolution, it is still an important way to increase yield and resistance by reducing or reactivating stem carbohydrates and increasing ovary or grain allocation during the critical growth period.

In summary, some promising strategies, such as rapid environmental response, optimized ear traits, and specific gene regulation in certain tissues or circumstances, aimed at optimizing the C economy within plant systems and increasing C flow to sink organs, have achieved synergistic improvements in yield and resistance/resilience. Based on these strategies, future research may lead to yield breakthroughs in multiple crops using different means (breeding and/or cultivation).

## Concluding thoughts

In conclusion, systemic improvement of crops should include the synergistic promotion of C fixation, transport, and utilization. Although the photosynthetic capacity of the field population of modern varieties is believed to be relatively high, as indicated by the traits of canopy leaf area, stay green, and stress tolerance, further improvements in photosynthetic efficiency and coupling with nutrients (N, P, and others) are still needed. One of the keys to high yields in the field is the efficient and economical use of photosynthetically produced assimilates. Unlike currently common adaptation strategies (for example, less C allocation under drought, seed abortion due to insufficient C supply, etc.), the C economy of future “smart crops” needs to be well designed, and the key lies in the flexible adjustment of C flow according to tissue needs at specific growth periods, aiming to improve yield and/or resilience. Both breeding and cultivation methods as well as crop physiology should be considered. In particular, understanding how a field crop manipulates its C economy is the basis for precise control. A recent study found that rapid phosphorylation of SWEET11 and 12 in *Arabidopsis* promotes carbohydrate transport to the roots during drought (Chen et al., 2022a). However, root C allocation and adjustment in crops have received limited attention. Hence, further research is needed to determine which tissue(s) should be stimulated during the growth process in the face of changing environments. Currently, some appropriate strategies, such as specifically overexpressing the TPP gene in maize and spraying T6P precursors after flowering or seedling drought in wheat, have been developed and applied (Griffiths et al., 2016; Oszvald et al., 2018). It is believed that in the future, “smart crops” created through means such as targeted gene editing, or “smart cultivation systems” based on precise C regulation will significantly contribute to the actual yield improvement.

## Author contributions

X-GL and ZG drafted and edited the manuscript and figure. XXF surveyed and analyzed the literature. X-GL and X-MC revised the manuscript. SS and S-LZ conceived the study, contributed to drafting, and edited both the text and figure. X-MC did a lot of work in the revision of the paper, including language & logic revision, re-creation and addition of figures. All authors contributed to the article and approved the submitted version.
